# Impacts of Synergy-505 on the Functional Response and Behavior of the Reduviid Bug, *Rhynocoris marginatus*

**DOI:** 10.1673/031.010.18701

**Published:** 2010-10-28

**Authors:** D. P. Ambrose, S. J. Rajan, J. M. Raja

**Affiliations:** Entomology Research Unit, St. Xavier's College (Autonomous), Palayankottai 627 002, India

**Keywords:** biocontrol agent, mating behavior, predatory behavior

## Abstract

The impact of the insecticide, Synergy-505 (chlorpyrifos 50% and cypermethrin 5% E.C), on the functional response, predatory behavior, and mating behavior of a non-target reduviid, *Rhynocoris marginatus* (Fabricius) (Hemiptera: Reduviidae), a potential biological control agent, were studied. Though both normal and Synergy-505-exposed *R. marginatus* exhibited Holling's type II curvilinear functional response, Synergy-505 caused a less pronounced type II functional response with reduced numbers of prey killed, attack rate, searching time, and prolonged handling time in 4th and 5th nymphal instars and adult males and females reflecting reduced predatory potential. Synergy-505 also delayed the predatory and mating events. The impacts of Synergy-505 on functional response, predatory behavior, and mating behavior were more evident at higher concentrations of Synergy-505.

## Introduction

Widespread and indiscriminate use of synthetic insecticides has resulted in undesirable ecological changes such as development of resistance in insects, resurgence of sucking pests, destruction of residues in or on soil and plant produce, risks to human beings, and harmful effects to animal health besides the effects of environmental pollution (Mahapatro and Gupta 1998).

Although the majority of modern synthetic insecticides are detrimental to beneficial insects, including natural enemies of crop pests, the effects vary from one insecticide to another and among different non-target beneficials (George and Ambrose 1998). Thus, screening of insecticides becomes imperative to safeguard non-target beneficials from the hazardous effects of insecticides (Ambrose 2001; Claver et al. 2003). *Rhynocoris marginatus* (Fabricius) (Hemiptera: Reduviidae) is one such predator that voraciously predates on various economically important insect pests (Ambrose 1999; George and Ambrose 2004). Although, the insecticidal impacts on biological and haematological parameters of reduviid predators have been studied (George and Ambrose 1999a, b, 2000, 2004), their impact on functional response, predatory behavior, and mating behavior have been neglected. Such an understanding of the sublethal effects of insecticides would enable selection of soft insecticides to protect beneficials and thereby improve the IPM. Such studies are very limited even in the field of agriculture (Ambrose 2001).

## Materials and Methods

Adults of *R. marginatus* were collected from Muthurmalai Scrub Jungle (altitude 125.33 MSL, latitude 77°° 21′? and 8°° 7′? N), Tirunelveli district, Tamil Nadu, South India. They were reared in the laboratory (28 –– 34°° C; 12:12 ±± 1 h L:D; 65––70 RH) in plastic containers (16 ×× 11.5 ×× 4 cm) feeding on larvae of the rice moth *Corcyra cephalonica* (Stainton) (Lepidoptera: Pyralidae).

Preliminary experiments were carried out to find the LC_50_ values, and 0.040% was found to be the optimum toxicity level of Synergy-505 (chlorpyrifos 50% and Cypermethrin 5% E.C). LC_50_ of 48 h duration was taken as one toxic unit and 1/10 the value of the 48 h LC_50_ of insecticide was considered as sublethal concentration (Croft, 1990). Sublethal concentration of insecticide was applied with a micropipette on 1 ×× 1cm size of absorbent papers and placed in the rearing containers. 30 laboratory reared fourth nymphal instars were reared in separate plastic containers (16 ×× 11.5 ×× 4.0 cm) with Synergy-505 applied absorbent papers as test individuals, and another 30 nymphal instars were reared with water applied absorbent papers as the control. Both Synergy-505- exposed and control sets of nymphal instars were allowed to grow up to adults.

The functional responses of one day-old control and Synergy-505-exposed 4^th^ and 5^th^ nymphal instars and adults to the larvae of *C. cephalonica* (0.8 to 1.2cm long) were studied in plastic containers (16 ×× 11.5 ×× 4 cm) at different prey densities (1, 2, 4, 8 and 16). The prey was first introduced into the experimental containers and was allowed to settle. After 30 min, a predator was introduced into the experimental container. The number of prey killed was continuously monitored, and fresh prey were introduced to replace the killed prey. After every 24 h, the prey consumed was counted. Eight replicates were maintained for each category and observations were continuously made for 6 days. Regression analysis (Daniel 1987) was carried out to determine the relationship between the prey density and the number of prey consumed, searching time, attack ratio, and handling time.

The impact of Synergy-505 on the predatory and mating behaviors of *R. marginatus* were studied by comparing the time durations taken for predatory events such as arousal, approach, capturing, paralyzing, and sucking; and mating events such as arousal, approach, and copulation in control and Synergy-505-exposed test individuals.

## Results and Discussion

The 50% lethality concentration (LC_50_) values, upper and lower fiducial limits, and toxicity of Synergy-505 on *R. marginatus* at 24, 48, 72, and 96 h durations are presented in Table 1, which shows that as the duration of Synergy-505 exposure was increased, the percentage of LC_50_ values and the upper fiducial limit decreased. The relative toxicity increased from 1.0 to 2.13 when exposure duration was increased from 24 to 96 h. Similar effects were also reported for a cypermethrin exposed to a reduviine reduviid, *Acanthaspis pedestris* Ståål (Claver et al. 2003), and monocrotophos, dimethoate, and quinalphos (George and Ambrose, 2004), and for methyl parathion, endosulfan (George and Ambrose 2006), and cypermethrin exposed-*R*. *marginatus* (Ambrose et al. 2007).

**Table 1.**
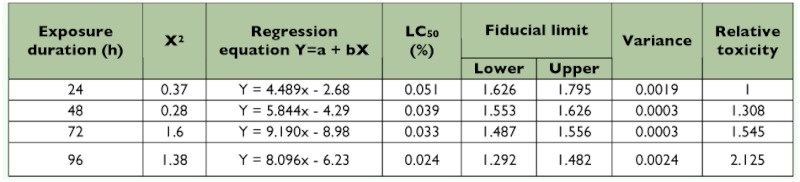
Toxicity of Synergy-505 to Rhynocoris marginatus (n = 30; d.f. = 3).

### Functional response

**Figure 1.**
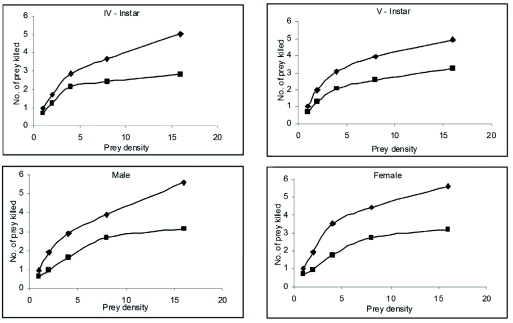
Functional response curves of control (triangle) and Synergy-505 (square) exposed 4^th^ and 5^th^ nymphal instars and adult males and females *Rhynocoris marginatus* at different prey densities. High quality figures are available online.

Control *R. marginatus* responded to increasing prey density by killing a higher number of prey than were killed at lower prey densities and thus exhibited Holling's type II curvilinear functional response (Holling 1959). The number of prey killed by the individual predator increased as the prey density (x) was increased from one prey per predator to 16 prey per predator. This was further confirmed by the positive correlations obtained between the prey density and prey killed for the 4^th^ and 5^th^ nymphal instars and adult males and females (y = 1.263 + 0.253x, r = 0.956; 1.502 + 0.238x, r = 0.928; 1.283 + 0.285x, r = 0.970 and 1.549 + 0.280x, r = 0.922; respectively). A similar functional response was observed in *A. pedestris* (Ambrose and Sahayaraj 1996; Claver et al. 2003), *Rhynocoris fuscipes* (Fabricius) (Ambrose and Claver 1995; Claver and Ambrose 2002), *Rhynocoris longifrons* Ståål (Claver et al. 2002), *Coranus spiniscutis* Reuter (Claver et al. 2004), and *Acanthaspis quinquespinosa* (Fabricius) (Ambrose et al. 2008). Though such positive correlations between the prey density and prey killed were also obtained for the Synergy-505-exposed 4^th^ and 5^th^ nymphal instars and adult males and females (y = 1.101 + 0.122x, r = 0.863; 1.008 + 0.154x, r = 0.927; 0.785 + 0.165x, r = 0.933 and 0.821 + 0.116x, r = 0.932; respectively), they exhibited reduced rates of predation (Tables 2––5 and Figure 1).

**Table 2.**
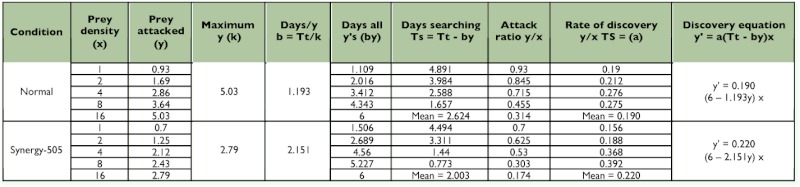
Functional response values for control and Synergy-505 exposed fourth nymphal instars of *Rhynocoris marginatus* to *Corcyra cephalonica* larvae for 6 days (n=12).

**Table 3.**
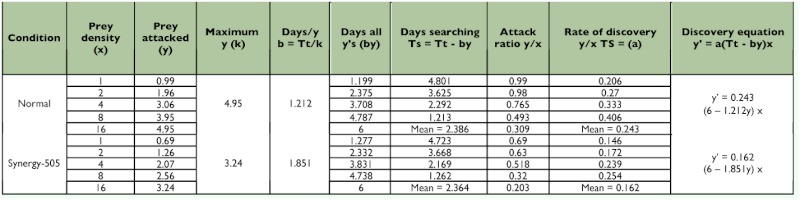
Functional response values for control and Synergy-505 exposed fifth nymphal instars of *Rhynocoris marginatus* to *Corcyra cephalonica* larvae for 6 days (n=12).

**Table 4.**
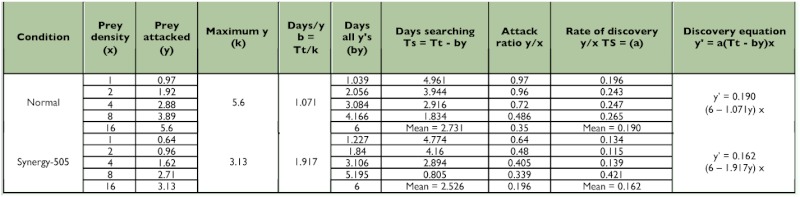
Functional response values for control and Synergy-505 exposed adult male *Rhynocoris marginatus* to *Corcyra cephalonica* larvae for 6 days (n=12).

**Table 5.**
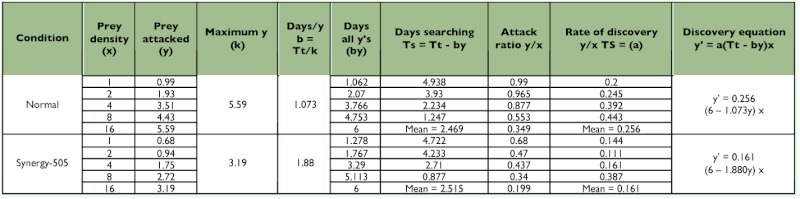
Functional response values for control and Synergy-505 exposed adult female *Rhynocoris marginatus* to *Corcyra cephalonica* larvae for 6 days (n=12).

The searching time decreased as the prey density was increased in both control and Synergy-505-exposed *R. marginatus* as evidenced by the negative correlations obtained between prey densities and the searching time for control (y = 4.493 - 0.302x, r = - 0.956; 4.179 - 0.289x, r = - 0.928; 4.625 - 0.306x, r = - 0.970 and 4.338 - 0.301x, r = - 0.922) and Synergy-505-exposed (3.631 0.262x, r = - 0.863; 4.133 - 0.285x, r = - 0.926; 4.493 - 0.317x, r = - 0.933 and 4.454 0.313x, r = - 0.932) 4^th^ and 5^th^ nymphal instars and adult males and females, respectively. But Synergy-505-exposed life stages of *R. marginatus* searched their prey quickly, and this reduction in the searching time was gradually reduced as the life stages grew (Tables 2––5). However, the cypermethrin-exposed *A. pedestris* took a longer time to search its prey due to insecticide repellency in searching behavior (Claver et al. 2003) as reported for several other natural enemies belonging to Aphelinidae, Syrphidae, and Trichogrammatidae (Ambrose 2001).

The maximum predation represented by k values was found restricted to high prey density in both control and Synergy-505-exposed life stages of *R. marginatus.* Prey density facilitated the predator to spend less time on its prey, and to utilize all its time attacking and consuming. The k value for control 4^th^ and 5^th^ nymphal instars and adult males and females were 5.03, 4.95, 5.60, and 5.59, respectively. Synergy-505-exposed 4^th^ and 5^th^ nymphal instars and adult males and females exhibited comparatively low predation rates as evidenced by low k values of 2.79, 3.24, 3.13, and 3.19, respectively (Tables 2––5). Similar insecticide-affected k values were observed for many arthropod beneficials (Croft 1990) and cypermethrin-exposed *A.* *pedestris* (Claver et al. 2003).

In both control and Synergy-505-exposed life stages of *R. marginatus* the highest attack ratios were found at 1 and 2 prey per predator densities and the lowest attack ratio at 16 prey per predator density and for both control (y = 0.904 - 0.041x, r = - 0.954; 1.000 - 0.047x, r = - 0.957; 0.962 - 0.042x, r = - 0.936 and 1.026 - 0.045x, r = - 0.973) and Synergy-505-exposed (0.682 - 0.035x, r = - 0.959; 0.672 0.032x, r = - 0.955; 0.566 - 0.025, r = - 0.921 and 0.588 - 0.026x, r = - 0.904) 4^th^ and 5^th^ nymphal instars and adult males and females, respectively (Tables 2––5). It is presumed that the predator spent less time on searching activities that might have caused a perceptive decline in the attack ratio until hunger was established. Such an indirectly proportional relationship between the attack ratio and prey density was earlier reported for several other reduviids (Ambrose 1999; Ambrose et al. 2000, 2008; Claver et al. 2003). The attack rate depends upon several component parameters, such as the rate of prey encounter, the probability that the prey will be attacked when encountered, and the probability that an attack will result in capture (Thompson 1975; Bailey 1986; Spitze 1985; Getty and Pulliam 1991).

Though the handling time (time taken by the predator to handle one host) decreased as the prey density increased in both control and Synergy-505-exposed *R. marginatus*, it was considerably prolonged in 4^th^ and 5^th^ nymphal instars and adult males and females from 1.193, 1.212, 1.071, and 1.073 min to 2.151, 1.851, 1.917, and 1.880 min, respectively (Tables 2––5). The present findings are in close agreement with those of cypermethrin-exposed *A. pedestris* (Claver et al. 2003). The resting time of the predator in between prey handling was longer at low prey density than at higher prey density.

There was a negative correlation between the rates of discovery and prey density in control (y = 0.274 - 0.013x, r = - 0.721; 0.334 0.014x, r = - 0.584; 0.277 - 0.014x, r = - 0.584; 0.277 - 0.014x, r = - 0.789 and 0.344 0.014x, r = - 0.500) as well as Synergy-505-exposed (y = 0.292 - 0.01x, r = - 0.431; 0.226 - 0.010x, r = - 0.627; 0.192 - 0.005x, r = 0.192 and 0.196 - 0.005x, r = - 0.253) 4^th^ and 5^th^ nymphal instars and adult males and females, respectively. But Synergy-505 reduced the rates of discovery at all prey densities in 5^th^ nymphal instar and adult males and females, and only at prey densities of 1 and 2 in 4^th^ nymphal instar (increased at 4 and 8 prey densities) (Tables 2––5). In *A. pedestris* cypermethrin rate of discovery decreased only at a prey density of 4 due to its decreased feeding rate (Claver et al. 2003).

### Predatory behavior

The act of arousal was delayed from 0.28 ±± 0.06 to 0.39 ±± 0.06, 0.26 ±± 0.07 to 0.46 ±± 0.13, and 0.31 ±± 0.03 to 0.76 ±± 0.26 min in the 4^th^ and 5^th^ nymphal instars and adults, respectively due to Synergy-505-exposure (Table 6).

Synergy-505 also prolonged the act of approach from 0.04 ±± 0.06 to 0.12 ±± 0.03, 0.02 ±± 0.01 to 0.06 ±± 0.02, and 0.16 ±± 0.01 to 0.20 ±± 0.04 min in 4^th^ and 5^th^ nymphal instars and adults, respectively. As observed for arousal and approach, Synergy-505 also delayed prey capturing in 4^th^ and 5^th^ nymphal instars and adults from 0.13 ±± 0.03 to 0.21 ±± 0.09, 0.11 ±± 0.02 to 0.14 ±± 0.06, and 0.38 ±± 0.07 to 0.45 ±± 0.15 min suggesting poor predatory efficiency due to Synergy-505-exposure (Ambrose 2001).

Synergy-505 also prolonged paralysing from 0.17 ±± 0.06 to 0.31 ±± 0.15, 0.15 ±± 0.17 to 0.21 ±± 0.10, and 0.18 ±± 0.06 to 0.36 ±± 0.07 min in 4th and 5th nymphal instars and adults, respectively as observed by Ambrose (1999, 2001).

Synergy-505 further delayed the act of piercing and sucking from 14.50 ±± 2.43 to 22.67 ±± 5.37, 13.83 ±± 3.89 to 19.17 ±± 5.58, and 16.50 ±± 2.98 to 20.67 ±± 5.73 min in 4^th^ and 5^th^ nymphal instars and adults. Such poor sucking efficiency as a function of insecticide exposure was reported for other reduviids (Ambrose 1999, 2001; Claver et al. 2003).

Similar observations of delayed predatory acts were reported by Claver et al. (2003) in cypermethrin-treated *A. pedestris.* Moreover, Synergy-505-exposed *R.* *marginatus* exhibited reduced food intake and often spitted watery saliva, as reported by Ambrose and George (1998) in monocrotophos-treated *A. pedestris.* The delayed predatory acts could be attributed to decreased movements due to malformed legs as a function of Synergy-505-exposure as observed by French-Constant and Vickerman (1985) in cypermethrin- and deltamethrin-exposed *Forficula auricularia.*

### Mating behavior

The Synergy-505 prolonged the time taken for arousal for mating. For instance, control individuals took 0.082 ±± 0.01 min to arouse whereas Synergy-505-exposed individuals took 0.315 ±± 0.09 min. The act of approach was also delayed from 0.33 ±± 0.05 to 0.58 ±± 0.11 min. As observed for arousal and approach, Synergy-505 also prolonged the duration of copulation from 33.50 ±± 8.94 to 21.67 ±± 7.23 min. The total duration of the mating (34.21 ±± 9.00 min) in control individuals was prolonged to 22.56 ±± 7.43 min in Synergy-505-exposed test individuals (Table 7).

**Table 6.**
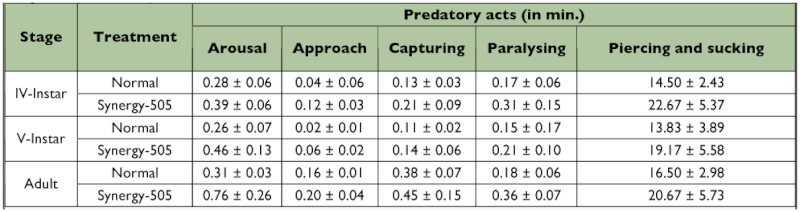
Chronological analysis of sequential acts of predatory events in normal and Synergy-505 exposed *Rhynocoris marginatus* (n = 6, ±± SD).

**Table 7.**
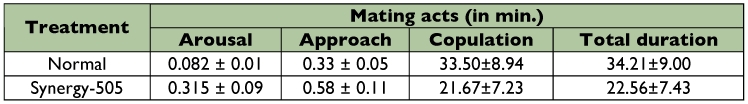
Chronological analysis of sequential acts of mating events in normal and Synergy-505 exposed *Rhynocoris marginatus* (n = 6, ±± SD).

Synergy-505-exposedmating partners not only showed significant deviations in terms of durations for each sequential act of mating from those of the control *R. marginatus*, but also failed to achieve genital connection. Such behavior was attributed to the inhibitory effects on various physiological processes (Ambrose and George 1998; Claver et al. 2003).

## Conclusion

Although the field concentration of (40µµl) of Synergy-505 did not immediately kill non-target predators like *R. marginatus*, it affected their functional response events such as number of prey attacked, attack ratio and rate of discovery and prolonged the predatory, and mating events. Hence, the results of the present study suggest that the usage of Synergy-505 is not advisable for a crop environment where beneficials like *R. marginatus* are found or incorporated as a biocontrol constituent in the integrated pest management program.

## References

[bibr01] Ambrose DP (1999). Assassin bugs.. New Hampshire, U.S.A: Science Publishers and New Delhi, India.

[bibr02] Ambrose DP (2001). Friendly insecticides to conserve beneficial insects.. *Zoos' Print Journal*.

[bibr03] Ambrose DP, Claver MA (1995). Functional response of *Rhynocoris fuscipes* Fabricius (Heteroptera: Reduviidae) to *Riptortus clavatus* Thunberg (Heteroptera: Alydidae).. *Journal of Biological Control*.

[bibr04] Ambrose DP, Claver MA, Mariappan P (2000). Functional response of *Rhynocoris*
*marginatus* (Heteroptera: Reduviidae) to two pests of pigeonpea (*Cajanus cajan*).. *Indian Journal of Agricultural Sciences*.

[bibr05] Ambrose DP, George PJE (1998). Comparative toxicological effects of monocrotophos to the third nymphal instars and the adults of *Acanthaspis pedestris* Ståål, a potential biocontrol agent (Insecta: Heteroptera: Reduviidae).. *Indian Journal of Environmental Sciences*.

[bibr06] Ambrose DP, Nambirajan SP, Ravichandran B (2007). Impact of Cypermethrin on the biology and life table parameters of a nontarget biological control agent *Rhynocoris marginatus* (Fabricius) (Hemiptera: Reduviidae).. *Hexapoda*.

[bibr07] Ambrose DP, Micheal Raja J, Jesu Rajan S (2008). Functional response of *Acanthaspis quinquespinosa* (Fabricius) (Hemiptera: Reduviidae) on *Coptotermes heimi* (Wasmann).. *Journal of Biological Control*.

[bibr08] Ambrose DP, Sahayaraj K (1996). Long term functional response of the reduviid predator *Acanthaspis pedestris* Stal (Heteroptera Reduviidae) in relation to its prey P*ectinophora gossypiella* Saunders, (Lepidoptera: Noctuidae) density.. *Hexapoda*.

[bibr09] Bailey PCE (1986). The feeding behaviour of a sit and wait predator, *Ranatra dispar* (Heteroptera: Nepidae) the combined effect of food deprivation and prey size on the behavioural components of prey capture.. *Ethology*.

[bibr10] Claver MA, Ambrose DP (2002). Functional response of the predator, *Rhynocoris fuscipes* (Heteroptera: Reduviidae) to three pests of pigeonpea (*Cajanus cajan*).. *Shashpa*.

[bibr11] Claver MA, Muthu MSA, Ravichandran B, Ambrose DP (2004). Behaviour, prey preference and functional response of *Coranus spiniscutis* Reuter, a potential predator of tomato insect pests.. *Pest Management in Horticultural Ecosystems*.

[bibr12] Claver MA, Ramasubbu G, Ravichandran B, Ambrose DP (2002). Searching behaviour and functional response of *Rhynocoris longifrons* (Ståål) (Heteroptera: Reduviidae), a key predator of pod sucking bug, *Clavigralla gibbosa* Spinola.. *Entomon*.

[bibr13] Claver MA, Ravichandran B, Khan MM, Ambrose DP (2003). Impact of cypermethrin on the functional response, predatory and mating behaviour of a non-target potential biological control agent *Acanthaspis pedestris* (Ståål) (Het., Reduviidae).. *Journal of Applied Entomology*.

[bibr14] Croft BA (1990). Arthropod biological control agents and pesticides..

[bibr15] Daniel WW (1987). Biostatistics: A foundation for analysis in the health sciences..

[bibr16] French-Constant RH, Vickerman GP (1985). Soil contact toxicity of insecticides to the European earwig *Forficula auricularia* (Dermaptera).. *Entomophaga*.

[bibr17] George PJE, Ambrose DP (1998). Effect of insecticides on the post-embryonic development in *Rhynocoris marginatus* (Fabricius) (Heteroptera: Reduviidae).. *Journal of Biological Control*.

[bibr18] George PJE, Ambrose DP (1999A). Impact of insecticides on the biochemical constituents in a non-target harpactorine reduviid, *Rhynocoris fuscipes* (Fabricius) (Heteroptera: Reduviidae).. *Shashpa*.

[bibr19] George PJE, Ambrose DP (1999B). Insecticidal impact on the post-embryonic development of *Rhynocoris kumarii* Ambrose and Livingstone (Het., Reduviidae).. *Journal of Applied Entomology*.

[bibr20] George PJE, Ambrose DP (2000). Impact of five insecticide on the diferential and the total haemocyte counts of *Rhynocoris marginatus* (Fabricius) (Insecta: Heteroptera: Reduviidae).. *Indian Journal of Environmental Sciences*.

[bibr21] George PJE, Ambrose DP (2004). Insecticidal impact on the life table parameters of a harpactorine reduviid predator *Rhynocoris marginatus* (Fabricius), Heteroptera.. *Entomologia Croatica*.

[bibr22] George PJE, Ambrose DP (2006). Effect of insecticides on the intrinsic rate of natural increase of *Rhynocoris marginatus* (Fabricius).. *Journal of Biological control*.

[bibr23] Getty T, Pulliam HR (1991). Random prey detection with pause travel search.. *American Naturalist*.

[bibr24] Holling CS (1959). Some characteristics of simple type of predation and parasitism.. *Canadian Entomologist*.

[bibr25] Mahapatro GK, Gupta GP (1998). Pesticide induced resurgence.. *Pestology*.

[bibr26] Spitze K (1985). Functional response of an ambush predator *Chaoborus americanus* predation on *Daphnia pulex*.. *Ecology*.

[bibr27] Thompson DJ (1975). Toward a predator-prey model incorporating age structure: the effects of predator and prey size on the predation of *Daphnia magma* by *Ischnura elegans*.. *Journal of Animal Ecology*.

